# Unusual manifestations of visceral leishmaniasis in children: a case series and its spatial dispersion in the western region of São Paulo state, Brazil

**DOI:** 10.1186/s12879-018-3652-1

**Published:** 2019-01-18

**Authors:** Luiz Euribel Prestes-Carneiro, Patricia Rodrigues Naufal Spir, Mateus Fontanesi, Karen Gabriella Pereira Garcia, Francisco Assis da Silva, Edilson Ferreira Flores, Dewton de Moraes Vasconcelos

**Affiliations:** 1 Infectious Diseases and Immunology Department, Oeste Paulista University, Presidente Prudente, São Paulo, Brazil; 2 Pediatrics Department, Oeste Paulista University, Presidente Prudente, São Paulo, Brazil; 3 Imunodeficiencies Outpatient Clinic, Pediatrics Outpatient Clinic, Regional Hospital of Presidente Prudente, Presidente Prudente, São Paulo Brazil; 4 Statistics Department, Paulista State University, Presidente Prudente, São Paulo, Brazil; 5 Laboratory of Medical Investigation Unit 56, Hospital das Clınicas da Faculdade de Medicina da Universidade de São Paulo, São Paulo, São Paulo Brazil

**Keywords:** Visceral leishmaniasis, Children, Unusual manifestations, spatial dispersion

## Abstract

**Background:**

Visceral leishmaniasis (VL) is becoming endemic in São Paulo state, in the southeastern region of Brazil. Unusual manifestations with non-specific signs and symptoms may make diagnosis difficult and delay treatment, increasing the risk of severity and death, particularly in new endemic areas. There are few studies on patients with these characteristics in Brazil. We describe a case series of unusual manifestations of VL in children and its spatial dispersion in the western region of São Paulo state.

**Cases presentation:**

From 2009 to 2014, five clinical cases involving children treated in the Regional Hospital of Presidente Prudente (RH) were selected. Two patients had multiple relapses requiring liposomal amphotericin B; one patient had VL-cytomegalovirus-dengue co-infection and liver injury; one patient was diagnosed with X-linked agammaglobulinemia, a primary immunodeficiency; and one patient was diagnosed with VL-human immunodeficiency virus/acquired immunodeficiency syndrome (VL-HIV/AIDS) co-infection. Primary or secondary immunodeficiencies were found in four children, and associated viral infections were found in three children. Three patients were referred from other hospitals to RH. With regard to the geographic spread of VL, more cases were found in the northern area, in the epicenter of the infection where the first cases were registered, flowing south; a spatial-temporal occurrence was found.

**Conclusions:**

Primary and secondary immunodeficiencies and viral co-infectious should be considered among unusual manifestations of VL, especially in those with multiple relapses. Spatial-temporal occurrence was found. Thus, integrated actions and effective monitoring of the disease are needed to complement curative practices to stem the tide of the epidemic.

## Background

VL is considered a neglected disease, with a higher prevalence in subtropical and tropical regions. In 2013, in Latin America, Brazil harbored 96% of cases, with most of the infected individuals living in the northeast region. In recent years, the disease has been spreading fast and has been reported in humans or dogs in 26 Brazilian states, with 19 states reporting cases of autochthonous human VL [1]. In 1996, infected dogs and humans were found in Araçatuba in the northwest of São Paulo state, and in 2006 the disease reached the western region, with 45 municipalities and about 900,000 inhabitants [2, 3]. These municipalities comprise the Regional Health Assistance Network11 (RRAS11), located in Presidente Prudente’s mesoregion. In this region, from 2006, when the first human case was reported in Dracena, a city with high transmission rates, to 2017, 18 of the 45 municipalities reported 492 human cases and 27 deaths, a lethality rate of 5.5% [4, 5]. The vector of VL, *Lutzomyia longipalpis* was found in 34 municipalities and canine VL was found in 28 municipalities [5].

In endemic areas of developing countries, about 50% of individuals treated for VL may be children [6]. VL is a long-term febrile disease and the clinical signs are non-specific and characterized by fever, paleness, weight loss, increased abdominal volume, hepatosplenomegaly, and edema. In new endemic areas, because of the non-specific signs and symptoms, VL can be misdiagnosed with delay in treatment, increasing the severity of the disease [4]. There are few studies on patients with these characteristics in Brazil. In this scenario, tertiary public hospitals play a key role in the Unified Public Health System throughout Brazil. They support smaller hospitals and more complex treatment procedures are performed [4]. Spatial analytic techniques have been used as valuable tools in epidemiologic studies, particularly for infectious diseases, including leishmaniasis. They can denote the points of occurrence and the intensity of certain phenomenon, helping public health authorities in their strategies for control and preventive measures. Furthermore, they can predict the dispersion of parasites and vectors and the influence of the neighborhood in the spread of the disease [2, 3, 7]. Here, we describe a case series of unusual manifestations of VL in children and its spatial dispersion in the western region of São Paulo state, considered a new focus of VL in Brazil. Cases were selected based on unusual manifestations in the emergency room, unexpected evolution on treatment, or rare co-morbidities/co-infections.

### Case series presentation

#### Case 1

**Fig. 1:**
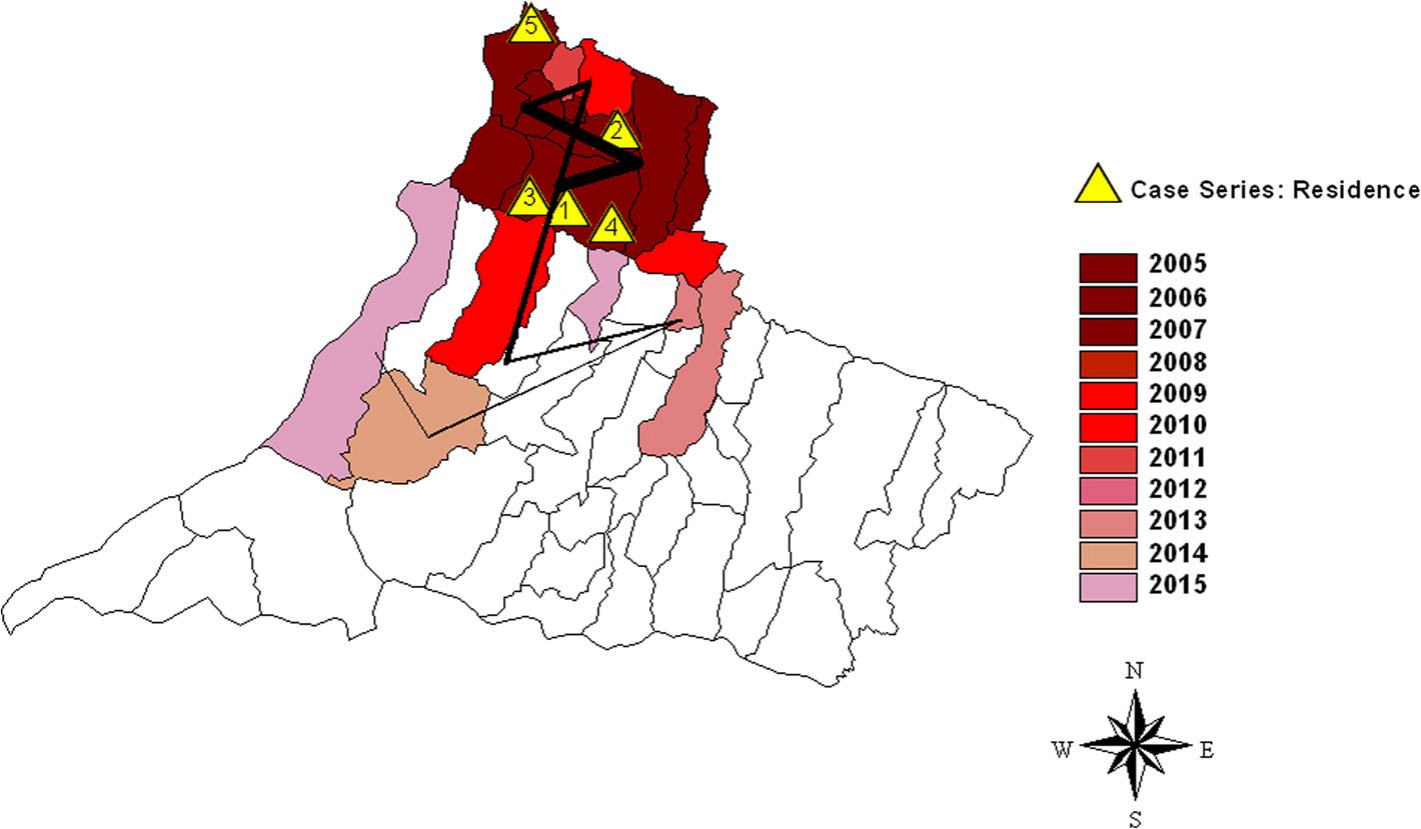
The flow and spatial dispersion of human visceral leishmaniasis from 2005 to 2015 in the RRAS11 mesoregion. The colors represent the year in which the first case of VL was detected and the numbers represent the municipality where the patients live or have been referred from. The lines represent the flow of the disease

On February 22, 2014, a 24-month-old girl referred from Dracena’s municipal hospital (Fig. 1, number 1) was admitted to RH and her parents related that she had had a fever for 10 days. In the previous 3 days, her health had worsened significantly with adynamia, pallor, inappetence, oliguria, and choluria. On systematic examination, she was lethargic, her skin was discolored (4^+^/4^+^), and hypoactive. A radiographic image on admission suggested pneumonia. Complete blood count showed marked anemia, thrombocytopenia, and a significant increase in hepatic enzymes (Table 1, column 1). Abdominal ultrasonography showed pronounced hepatosplenomegaly. Supported by her health status, laboratory and image examinations, and the fact that she lived in an endemic region, VL was suspected. The laboratory diagnostics (Table 1, column 1) recommended mainly by the Manual of Surveillance and Control of Visceral Leishmaniasis of São Paulo state [8–11] included direct parasitology consisting of the presence of amastigotes of *Leishmania* in bone marrow aspirate stained by Giemsa stain; a serological titer ≥1:80 in an indirect fluorescent antibody test (IFAT; Bio-Manguinhos/FIOCRUZ, Rio de Janeiro, Brazil); and in 2010, the rK39 rapid diagnostic test (Kalazar Detect, InBios, Seattle, Washington, USA) was implemented. She was sent to the pediatric intensive care unit (ICU) from the emergency department and treated with liposomal amphotericin B (5 mg/kg/day) for  5 days. Serology screening showed that she was positive for cytomegalovirus (CMV) antibodies IgG and IgM. At the end of VL treatment, she had severe liver injury, presenting hyperbilirubinemia, hypoalbuminemia, and increased liver and canalicular enzymes: alanine transaminase (ALT) 354 IU/mL; aspartate transaminase (AST) 401 IU/mL; alkaline phosphatase 993 mg/dL; γ-glutamyl transferase 1048 mg/dL; total bilirubin 5.77 mg/dL, leading to fecal acholia, jaundice, and hypoalbuminemia (2.7 g/dL). Because there was an outbreak of dengue fever in her home region, dengue was suspected and IgM serology was positive in two different samples. She remained in the pediatric ICU for 12 days and was discharged after 24 days. At discharge, the case was diagnosed as VL-CMV-dengue virus co-infection and liver injury, and she was followed for 1 year as an ambulatory patient with pediatric infectious diseases. In March, 2017, she was hospitalized with severe respiratory distress and renal failure, remaining in the pediatric ICU; she died 2 days later with a diagnosis of Brazilian spotted fever transmitted by ticks for which the causative agent is *Rickettsia rickettsii*.

**Table 1:** Laboratory parameters at presentation, clinical profile, and findings

	Clinical cases					Normal range
	1	2	3	4	5	
Hepatomegaly	Present	Present	Present	Present	Present	
Splenomegaly	Present	Present	Present	Present	Present	
Hemoglobin (g/dL)	7.3	10.4	7.9	8.9	9.7	10.5–14.5
Leukocytes (mm^3^)	8.910	12.600	4.150	7.570	4.690	6–17.5
Platelets (mm^3^)	70,000	160,000	48,000	934,000	18,000	150–400
AST (U/L)	126	16	44	21	15	0–35
ALT (U/L)	468	45	68	22	30	0–40
Diagnosis						
Bone marrow	P	P	P	P	P	
IFAT	P	ND	P	N	N	
rK32	N	ND	P	N	N	
Hospital days	24	8	15	7	71	

*AST* aspartate aminotransferase, *ALT* alanine aminotransferase, *P* positive, *IFAT* indirect fluorescent antibody test, *ND* not done, *N* negative; rK32, VL rapid test

#### Case 2

On May 28, 2009, a 13-month-old boy residing in Tupi Paulista municipality (Fig. 1, number 2) was admitted with fever, inappetence, and splenomegaly. On systematic examination, he was pale and febrile with a distended abdomen and hepatosplenomegaly. Because he lives in an endemic area, VL was suspected and bone marrow aspirate examinations were conducted according to the laboratory diagnostics recommended by the Manual of Surveillance and Control of Visceral Leishmaniasis of São Paulo state [8] (Table 1, column 2). At diagnosis, his laboratory results were as follows: total protein 6.8 g/dL, albumin 3.6 g/dL, globulin 3.2 g/dL, and A:G ratio of 1.1 with a 2-fold increase and 12-fold increase in IgG and IgE immunoglobulin levels, respectively. Serum protein electrophoresis revealed polyclonal hypergammaglobulinemia: total protein 7.3 g/dL, albumin 3.95 g/dL, alpha1-globulin 0.41 g/dL, alpha2-globulin 0.76 g/dL, beta1-globulin 0.43 g/dL, beta2-globulin 0.23 g/dL, gamma-globulin 1.53 g/dL (20.9%; reference 10.6%–18.8%). He was treated with liposomal amphotericin B (5 mg/kg/day) for 5 days with improvement in the symptoms and 50% reduction of splenomegaly. CMV IgG and IgM antibodies were detected in the serology screening. After 8 days, he was discharged with a diagnosis of VL-CMV co-infection. However, the patient had a relapse several times at 59 days, 79 days, and 156 days after discharge. On each recurrence, he was admitted to the pediatric ward and underwent the standard treatment for 5 days. After the last infection, he was treated with an HIV-positive immunosuppressed regimen (liposomal amphotericin B, 5 mg/kg/day, 1 day per month for 6 months). A primary immunodeficiency was investigated and immunophenotyping and lymphocyte proliferation were at normal levels (Table 2, column 1). At discharge, he was diagnosed as VL-CMV co-infection and treated with an immunosuppressed regimen. After treatment, immunoglobulin and serum protein electrophoresis showed normal levels. After this protocol, he did not relapse and was followed for 1 year as an ambulatory patient with pediatric infectious diseases.

**Table 2:** Immunological markers of the patients at presentation and at follow-up

Immunological findings	1	2	3
Immunophenotyping			
CD45/CD3 (1200–2600/mm^3^)	2397	3257	1617
CD45/CD3/CD4 (650–1500/mm^3^)	1018	2079	1062
CD45/CD3/CD8 (370–1100/mm^3^)	1167	1051	499
CD45 (3600–9800/mm^3^)		6968	1200
CD45/CD19 (720–2600/mm^3^)	1553	3133	0
CD45/CD3^−^/CD16^+^ (100–480/mm^3^)	153	277	80
Lymphocyte proliferation			
Phytohemagglutinin (SI) (18.28–343.00)	25	33.0	61.2
Pokeweed (SI) (8.42–107.40)	7.0 ↓	35.2	11.5
OKT3 (SI) (15.62–219.30)	21	7.6 ↓	26.2
Cytomegalovirus (SI) (3.04–56.6)	ND	2.3 ↓	3.1
Response to vaccination			
Anti-HBs	ND	↓	↓
Anti-tetanus	ND	↓	↓
Anti-pneumo 23 antibodies	ND	↓	↓
Immunoglobulins			
IgG (232–1411 mg/dL)	2940	1734	42
IgM (19–145 mg/dL)	118	39	29
IgA (0–83 mg/dL)	155	31	< 7
IgE (0–24 K/U/L)	289	55	< 7
IgG subclasses			
IgG1 (1700–9500 mg/L)	ND	4710	266
IgG2 (215–4400 mg/L)	ND	544	89
IgG3 (134–694 mg/L)	ND	310	99
IgG4 (4–1200 mg/L)	ND	210	21

*SI* stimulation index, *ND* not determined

#### Case 3

On November 13, 2014, a 14-month-old girl residing in Ouro Verde municipality (Fig. 1, number 3) was referred from Dracena’s municipal hospital; her parents related that she had been vomiting and had had a fever for 9 days. VL was suspected and bone marrow aspirates, IFAT and rK39 were conducted according to the laboratory diagnostics recommended by the Manual of Surveillance and Control of Visceral Leishmaniasis of São Paulo state [8] (Table 1, column 3). On systematic examination, her health status was regular, but she was pale (1+/4+), with a distended abdomen and hepatosplenomegaly (Table 1, column 3). She was treated with liposomal amphotericin B (5 mg/kg/day) for 5 days and was found to have severe anemia. Red blood cells (15 mL/kg/day) were transfused twice, and the patient was discharged 15 days later with a diagnosis of VL. However, the patient had a relapse several times at 98 days, 131 days, 156 days, and 171 days, with the same symptoms at different time intervals. On each recurrence, she was admitted to the pediatric ward and underwent the standard treatment for 5 days. After the last hospitalization (at 171 days), she was treated with an HIV-positive immunosuppressed regimen (liposomal amphotericin B, 5 mg/kg/day, 1 day per month for 6 months). A primary immunodeficiency was investigated and immunophenotyping showed normal levels, however a decrease in lymphoproliferation against mitogens and CMV antigen was found (Table 2, column 2). In the course of the relapse treatment, she had multiple upper airway infections (community-acquired pneumonia, tonsillitis, and sinusitis). Specific antibody responses to pneumococcal polysaccharide vaccine (representing a T cell-  independent response) and tetanus, diphtheria, and hepatitis B virus after vaccination (representing a T cell-dependent response) were investigated. The concentration of antibodies was below protective levels after vaccination. Furthermore, the patient showed a poor polysaccharide antibody response to a 23-valent pneumococcal vaccine. After completing the immunosuppressed regimen, she did not relapse, and she is being followed as an ambulatory patient with pediatric infectious diseases and immunodeficiencies.

#### Case 4

On November 23, 2010, a 8-month-old boy referred from Dracena’s municipal hospital (Fig. 3, number 4) was admitted to RH with fever, cough, and vomiting for the previous 5 days. On systematic examination, he was pale, febrile, dehydrated with tachydyspnea, distended abdomen, and hepatosplenomegaly. He was sent to the pediatric ICU and treated for community-acquired pneumonia. Because he lives in an endemic area, VL was suspected and bone marrow aspirate examinations were conducted according to the laboratory diagnostics recommended by the Manual of Surveillance and Control of Visceral Leishmaniasis of São Paulo state [8]. (Table 1, column 4). He was treated with liposomal amphotericin B (5 mg/kg/day) for 5 days. Immunoglobulin levels were determined, resulting in pan-hypogammaglobulinemia. Immunophenotyping and lymphocyte proliferation were investigated, and he was diagnosed as X-linked agammaglobulinemia (XLA). Patients with XLA have absent or reduced number of peripheral B cells and a profound deficiency in all immunoglobulin isotypes (Table 2, column 3). He was discharged after 20 days, and he is being followed as an ambulatory patient with immunodeficiencies with human IgG immunoglobulin (400 mg/kg) replacement at intervals of 28 days.

#### Case 5

In 2011, a 11-year-old girl, residing in Paulicéia municipality (Fig. 3, number 5), was admitted to RH (personal data [12]). She was AIDS-C3 with low levels of CD4, high viral load, severe diarrhea, oral and perineal candidiasis, severe thrombocytopenia, and protein-caloric malnourishment. She had sepsis and renal and cardiac failure. She was sent to the pediatric ICU, and because she lives in an endemic region, VL was suspected. Bone marrow aspirate examinations according to the laboratory tests recommended by the Manual of Surveillance and Control of Visceral Leishmaniasis of São Paulo state were conducted [8] (Table 1, column 5). Her symptoms improved significantly after administration of liposomal amphotericin B. However, on the 47th day of hospitalization, she had a relapse with thrombocytopenia and retreatment with liposomal amphotericin B and intravenous human IgG immunoglobulin was given. She was discharged after 71 days, diagnosed as VL-HIV/AIDS co-infection. The patient was lost to follow-up.

## Discussion and conclusions

American Tegumentar Leishmaniasis (AT) and VL are found in all regions of Brazil. The main causative agents of AT are *Leishmania* (*Leishmania*) *amazonensis*, *Leishmania* (*Viannia*) *guyanensis*, and *Leishmania* (*Viannia*) *braziliensis* which is responsible for the muco-cutaneous form in São Paulo state. Countrywide, *Leishmania infantum* (syn. *Leishmania chagasi*) is the causative agent of VL, and it is found especially in dogs (the main domestic reservoir), in cats and in humans [9–11]. Using different independent molecular methods, the genetic diversity of *L. infantum* isolates obtained from different endemic areas in Brazil revealed 18 very similar genotypes [11].

Despite the measures adopted by public authorities to control VL throughout São Paulo state, there is evidence that the disease is spreading at an alarming rate throughout the western region [4, 12, 13]. Since 2009, RH has been a reference center for the diagnosis and treatment of children thought to be infected with VL in the RRAS11 region. From January 2005 to December 2017, 157 children have been diagnosed and treated.

VL is an immune-mediated disease affecting mostly children because of their immature cell-mediated immune system, the cornerstone effector mechanism in VL infection, and older people as a result of senescence [4–12]. Among the patients selected for this study, four (80%) showed immune deregulation at diagnosis or during follow-up.

Case 1 involved co-infection with VL and dengue virus, both blood-borne diseases with a biological cycle in the liver and liver dysfunction. In patients infected with VL, a good prognosis is associated with reduction in the effects on the liver and spleen by 50% from onset to discharge and a decrease or normalization of the hepatic enzymes at the end of treatment [14, 15]. The cells of the reticuloendothelial system in the liver harbor VL parasites, and hepatomegaly is common. In India, functional derangement of the liver in individuals infected with VL was found to be common [16]. Surprisingly, in our patient, at the end of treatment for VL, she had severe liver injury, and dengue IgM serology confirmed VL-dengue virus co-infection. Liver involvement in acute dengue infection is frequently observed, leading to liver dysfunction. The severity depends on hypoxic injury as a result of decreased perfusion, the degree of viral load, and the deregulated immunologic lesions triggered by inflammatory immune mediators [17]. The spectrum of involvement ranges from asymptomatic increased levels of hepatic enzymes to acute liver failure. As in our patient, in children infected with dengue, increased AST/ALT levels, hepatomegaly, jaundice, and hypoalbuminemia are the main features. In Thailand and in Northern India, dengue was the major cause of acute liver failure in children with rates of 18.5 and 34%, respectively [17, 18]. However, to our knowledge, a VL-dengue co-infection has not been described previously.

Cases 2 and 3 were characterized by relapses with repeated treatments with liposomal amphotericin B. Following the Manual of Surveillance and Control of American Visceral Leishmaniasis of the State of São Paulo [8], the use of amphotericin B is restricted to a few special conditions. In 2006, in an effort to lower death rates from VL, especially in children, attributed to the use of pentavalent antimony as first-line therapy, São Paulo state recommended liposomal amphotericin B therapy for all children up to 10 years of age [19]. Relapse after treatment with liposomal amphotericin B is associated with increased lethality rates, poor prognosis, and disease severity among children [14, 20]. Little is known regarding the characteristics of apparently immunocompetent patients with VL who relapsed among different regions, treatment regimens, and study populations. In the Brazilian guidelines, there are no instructions on how to manage these patients. In Sudan, relapsed patients showed splenomegaly at diagnosis and at discharge [15, 21]; in Georgia, delay in the diagnosis for > 90 days, low levels of hemoglobin, and age < 1 year [22]; in India, male sex, aged 5–45 years, a smaller decrease in splenomegaly at discharge, and a shorter duration of symptoms before seeking treatment were strongly associated with relapse [14]. In patients with acquired immunodeficiency, mostly patients with HIV infection, the burden of VL infection and relapse is aggravated by impaired T cell number and function and, consequently, decreased T and B cell interaction [23, 24]. In an attempt to find the primary immunodeficiency that explained the increased number of relapses in our patients, immunophenotyping, lymphocyte proliferation, and humoral response against vaccines were conducted. A normal range of peripheral immune cells was found in both patients, however, patient 3 (Table 2, column 2) showed a decrease in lymphoproliferation against OKT3 (anti-CD3) mitogen and CMV antigen. Furthermore, she did not respond to T cell-dependent vaccines, anti-HBS and anti-tetanus, or to T cell-independent pneumo-23 polysaccharide vaccines. These results explain in part the repeated upper airways infections that affected the patient during treatment with liposomal amphotericin B in the relapsing period. Conversely, at onset of diagnosis of VL, patient 2 (Table 2, column 1) showed polyclonal gammopathy with increased levels of IgG and IgE and a slight hypergammaglobulinemia that quickly decreased to normal levels after treatment. Both patients recovered when the protocol for secondary prophylaxis to immunosuppressed patients was applied. Patients 2 and 3 required great effort from our team who contacted many other hospitals and doctors to discuss the drug and the duration of treatment to be adopted. Currently, a new protocol addressing these issues is being proposed in the state of São Paulo. Increasing rates of relapse against liposomal amphotericin B, the standard treatment for children, are occurring in our region. Of 157 children treated, 15 have relapsed (9.5%).

Case 4 arrived in the emergency room with fever, tachydyspnea, and cough, and community-acquired pneumonia associated with VL was diagnosed. Certainly, the undiagnosed primary immunodeficiency contributed to the severity of his condition. XLA deficiency is caused by mutations of the Bruton tyrosine kinase gene and is the most common form of severe inherited antibody deficiency. It is characterized by a fully penetrant X-linked recessive disorder with recurrent bacterial infections, including upper and lower airways infections, profound hypogammaglobulinemia, and marked decrease or absence of peripheral mature B cells [25]. As shown in Table 2, column 3, he exhibited normal levels of T cell subsets and lymphocyte proliferation against mitogens. However, total depletion of CD19+ B cells, no antibody response to vaccine antigens, representing a decrease in a B and T cell-dependent response, and hypogammaglobulinemia were found. He was not receiving intravenous immunoglobulin replacement therapy at the diagnosis of VL. To our knowledge, this is the first report of VL-XLA infection worldwide.

Case 5 represents an unwelcome synergy and was the first case of a child co-infected with HIV/AIDS and VL described in the western region of São Paulo state [12]. In Brazil, from January 1980 to June 2016, 842,710 cases of AIDS were reported, and the rate of detection of AIDS in children < 5 years of age has been used as a proxy indicator for monitoring the vertical transmission of HIV, with a local and countrywide reduction [26–28]. HIV-VL co-infection is considered an emerging disease in some developed and developing countries, with increasing rates since the beginning of the 1990s. The burden of the disease is fueled by geographic superposition of the two infections as a consequence of the urbanization of VL and the internalization of HIV infection [28, 29]. Previously, VL was restricted to rural areas in the northeastern region, responsible for 70–90% of cases, but since the 1980s, the formation of shantytowns with very poor living conditions favored important outbreaks in large Brazilian cities. Conversely, as a result of the countrywide spread of HIV infection in all social classes, outbreaks have moved from large urban centers to the smaller towns [28–30]. This new scenario represents a challenge for the control of HIV-VL co-infection [23]. In Brazil, HIV-VL co-infection has increased from 0.7% in 2001 to 8.5% in 2012 [23, 24]. HIV/AIDS is burdensome for children co-infected with VL, with decreasing anti-leishmania antibodies titers and therapeutic response, increasing susceptibility to severe hospital-acquired infections, greatly increased probability of relapse, and high mortality rates [12, 23, 24, 30]. The onset of clinical manifestations, diagnosis, and treatment of VL and discharge of the patient at 71 days was longer than usual in children treated in RH. In new endemic areas, lack of awareness of the occurrence of unusual manifestations may cause considerable difficulties in establishing the diagnosis [4, 12], and should be considered the reason for the delay in the diagnosis of VL in this case report.

In these selected case reports, 50% were associated with viral infections: CMV, dengue, and HIV. Co-infection of CMV with leishmaniasis produces a diagnostic dilemma because of the similarity of some clinical features and hematologic parameters, including fever, leukopenia, and thrombocytopenia [31]. Moreover, CMV infection has an immunomodulating and immunosuppressant effect on humoral and cellular immune responses, predisposing the patient to other infections. CMV may also be part of the super-infections reported in patients with VL [32].

Three (60%) of our patients were referred from other hospitals, and three (50%) were sent from the emergency room to the ICU because of worsening of their clinical status, reinforcing the role of tertiary hospitals in the Brazilian public health system and fueled by the severity of unusual manifestations of VL.

**Fig. 2:**
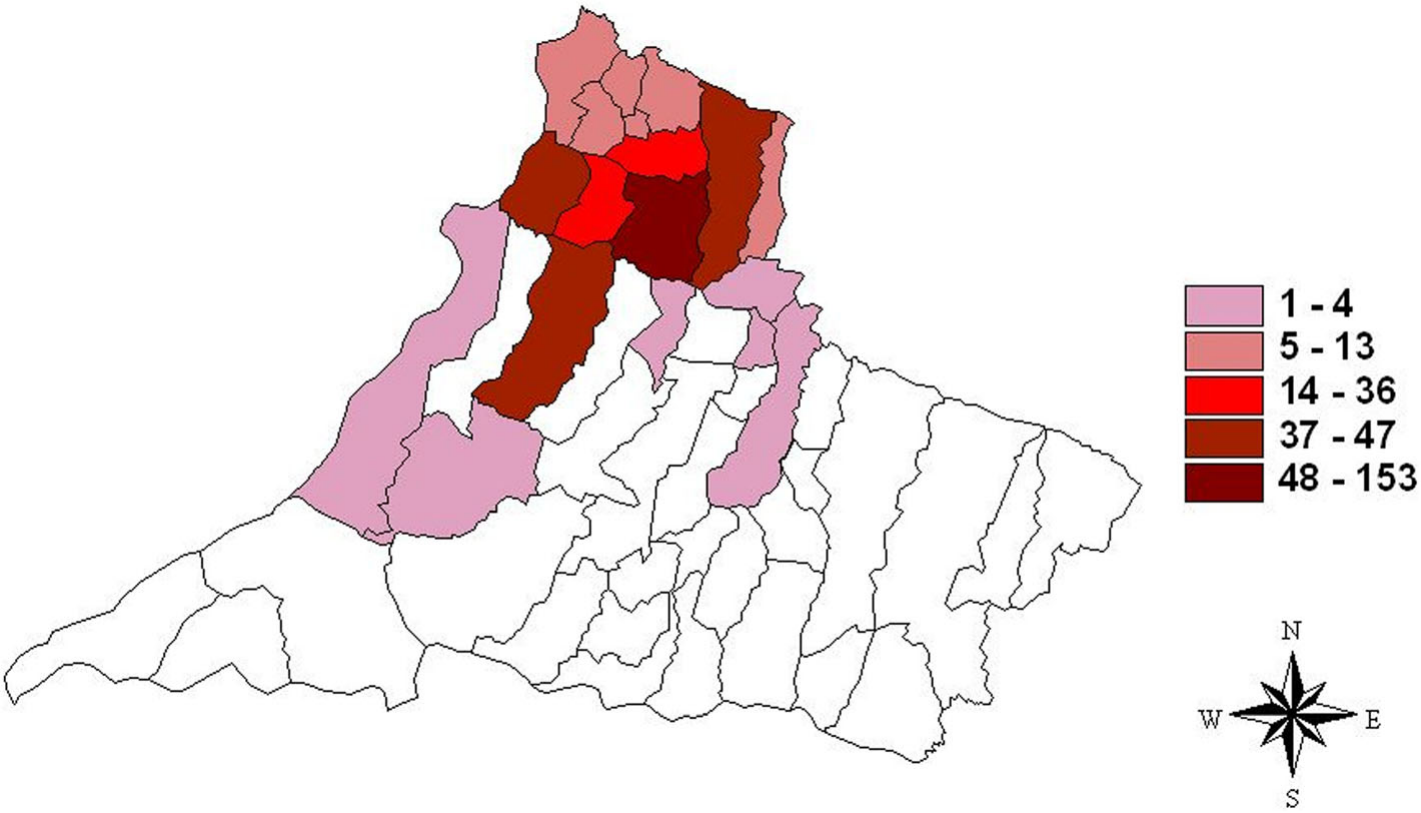
Geographical distribution of human visceral leishmaniasis in the RRAS11 mesoregion, between 2005 and 2015. The colors represent the accumulative numbers of cases

**Fig. 3:**
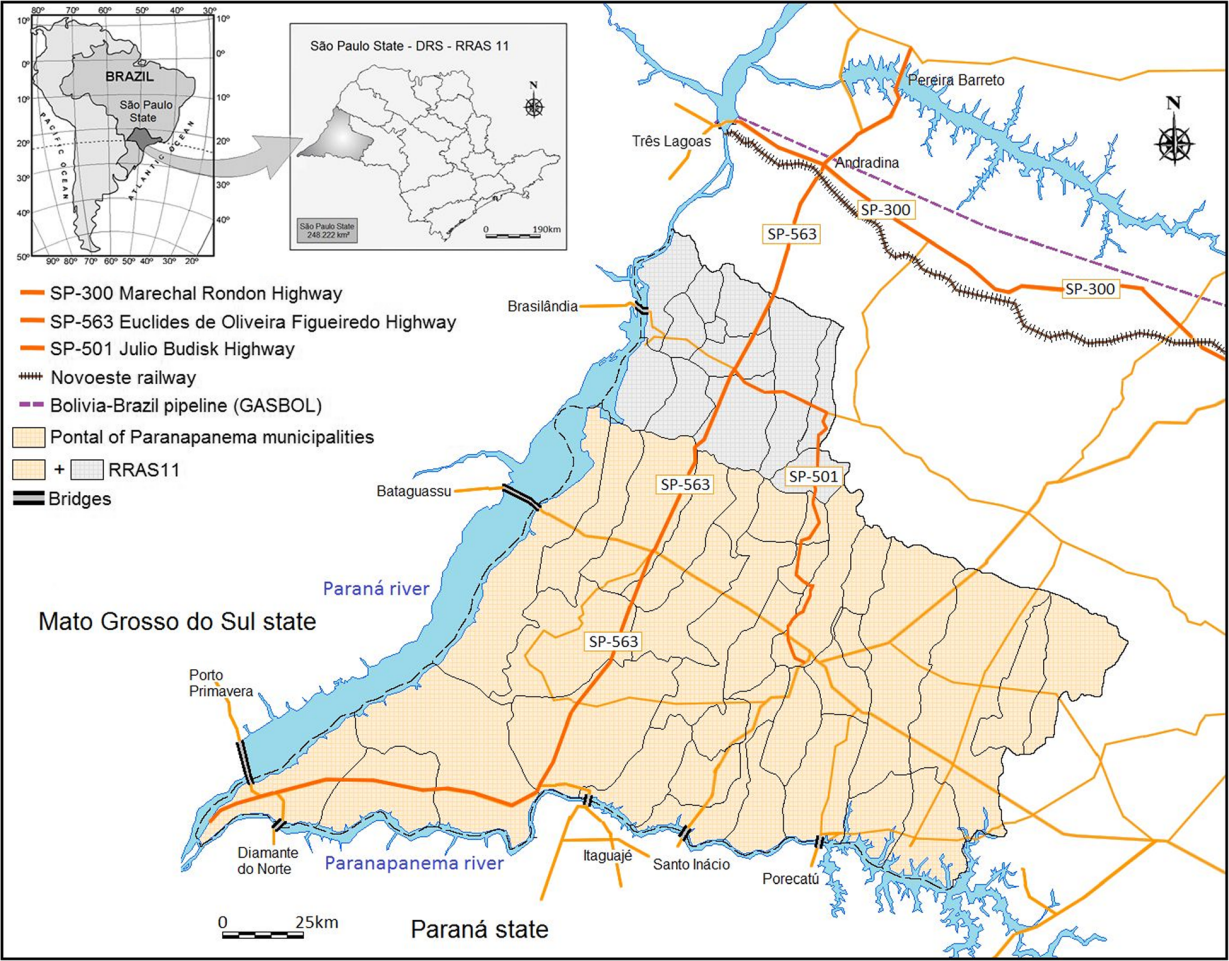
Geographic location of São Paulo state, Brazil, and the study area. The mesoregion of Presidente Prudente, within RRAS11, and the neighboring states of Mato Grosso do Sul (MS), and Paraná (PR) and the network of highways linking different municipalities. The 33 municipalities of Pontal of Paranapanema are in RRAS11. Paraná River on the border of MS and Paranapanema River on the border of PR with lakes supporting hydroelectric plants and bridges. Data were obtained from the Brazilian Institute of Geography and Statistics

São Paulo state has experienced a rapid spread of the vector and canine and human VL over the last two decades, and some regions, including the western region, are becoming new endemic areas in Brazil [4, 13]. In this region, the epicenter of the infection was in the northern part, with clusters of municipalities harboring higher numbers of cases (48–153), bordered by municipalities with a medium (14–36) and low number of cases (1–4), in 2005–2015 (Fig. 2). These municipalities, are linked by contiguity to the municipality of Andradina, outside RRAS11 and considered endemic by the Brazilian Ministry of Health since 2005 [4, 12, 13] (Fig. 3). All children in this report lived in municipalities in which the disease was first described in 2005–2006. Cases 1 and 4 lived in Dracena, case 2 in Tupi Paulista, case 3 in Ouro Verde, and case 5 in Paulicéia (Figs. 1 and 2). With the exception of Paulicéia, the other municipalities registered medium or high number of cases in 2005–2015 (Fig. 2). From Dracena, the disease spread toward the north and then toward the municipalities of Pontal of Paranapanema, the poorest and most undeveloped region of São Paulo, and toward Paraná state in the south (Figs. 1 and 3). Considering these results, a spatial-temporal geographic occurrence in the spread of VL was found in the western region of São Paulo state.

The reasons why VL is spreading in such a fast and alarming way in this region are not well understood. A complex array of factors may be fueling the epidemic and sustaining endemic transmission [4]. In São Paulo state, the association of the spread of VL throughout the area around the Marechal Candido Rondon Highway, the Bolivia-Brazil railway, and the gas pipeline construction (GASBOL), linking the endemic region of Mato Grosso state in direction of São Paulo, is overwhelming [3] (Fig. 3). Similarly, we suggest that in RRAS11, the dispersion of canine VL and human VL vectors is associated with the Euclides Figueiredo Highway as the primary axis, and the Julio Budinsky Highway as the secondary axis. Nevertheless, dissemination of the vectors of VL could be aggravated by the extensive watershed flowing into the three biggest rivers of southwestern and southern Brazil, with nine large lakes and a flooded area of 2384 mile^2^, supporting hydroelectric plants and bridges. These bridges link dozens of small towns and villages connected by a large number of highways (1480 miles). They support a daily flow of people, cars, animals, and goods linking endemic regions to VL-free areas (Fig. 3).

Some shortcomings should be mentioned when interpreting our data. This is a retrospective study over 10 years with selection bias as a result of different investigators. The protocol for VL diagnosis changed during the study period and immunophenotyping and lymphocyte proliferation were not available for all patients.

Primary and secondary immunodeficiencies and viral co-infections should be considered among unusual manifestations of VL, especially in those with multiple relapses. In this scenario, tertiary reference hospitals have a key role in the Brazilian public health system. In the geographic spread of VL, a higher number of cases were demonstrated in the northern part, in the epicenter of the area where the first cases were registered, flowing to the south and a spatial-temporal occurrence was found. Thus, integrated actions and effective monitoring of the disease are needed to complement curative practices to stem the tide of the epidemic.

## Abbreviations

ALT: Alanine transaminase
AST: Aspartate transaminase
CMV: Cytomegalovirus
HIV/AIDS: Human immunodeficiency virus/acquired immunodeficiency syndrome
ICU: Intensive care unit
RH: Regional Hospital of Presidente Prudente
RRAS11: Regional Health Assistance Network11
VL: Visceral leishmaniasis
XLA: X-linked agammaglobulinemia
